# The Life Cycle of the Parasitic Crustacean, *Lernanthropus latis* Yamaguti, 1954 (Copepoda: Lernanthropidae), on Marine-Cultured Fish, *Lates calcarifer*, from Setiu Wetland, Terengganu

**DOI:** 10.1155/2014/697134

**Published:** 2014-12-10

**Authors:** Nur Qamarina Abdul Khalid, Faizah Shaharoum-Harrison

**Affiliations:** ^1^Institute of Tropical Aquaculture, Universiti Malaysia Terengganu, 21030 Kuala Terengganu, Malaysia; ^2^Kenyir Research Institute, Universiti Malaysia Terengganu, 21030 Kuala Terengganu, Malaysia

## Abstract

Parasitic crustaceans of *Lernanthropus latis* were isolated from the host, the seabass, *Lates calcarifer*, obtained from a cage culture in Setiu Wetland, Terengganu. The adult females with egg were kept alive in vials containing 20 mL of filtered seawater and incubated at 30°C. The eggs were monitored every hour and the hatching periods were recorded. Three developmental stages were observed, namely, nauplii I, nauplii II, and infective copepodid. The infective copepodids were then transferred into a tank containing 60 litres of seawater with 150 fingerlings for infection purpose. One fish was sacrificed every 24 hr to inspect the next developmental stage. As a result, six more stages were obtained within 298 hrs starting from the infection day. The stages were known as fixed copepodid I, fixed copepodid II, fixed copepodid III, fixed copepodid IV, preadult, and adult. Parasitic *L. latis* takes a 483 hr period to complete a life cycle.

## 1. Introduction

Parasites are common in the animal kingdom, and they play significant roles in marine aquaculture [1].* Lernanthropus* De Blainnville, 1822, is the most widespread genus of the family Lernanthropidae which is considered to be a common genus of parasitic copepods on fishes. They attach to the gill filaments of its host by means of the piercing action of the antennae [2], assisted by maxillipeds and third legs [3]. The fourth pair of legs is modified, usually very long and apparently functionless. Most members are also characterized by the presence of plate-like structures and outgrowths of the fourth leg-bearing segment, often completely covering the posterior half of the body [4]. Occasionally, the parasitic copepods may injure their host by feeding on mucus and skin [5] and sucking the host's blood [6] especially those which attach to the gills. Such feeding activity can cause mechanical damage to the skin, fin erosion, and osmotic stress [7] and may result in death in extreme cases [8]. The combination actions of the antennae, maxilla, and maxilliped together with mandible of* Lernanthropus latis* which caused the damage were detected under light and scanning electron microscopies (SEM) [9]. The pathological effect such as erosion, haemorrhages, hyperplasia, and necrosis along the secondary lamellae of gill filaments of* L. latis* was observed with a great severity at the site of attachment. It is important to study the biology and life cycle due to the attachment location on the gill filaments and their blood-feeding habit which generates a threat to their host when outbreaks occur. Thus, some prophylactic measures to prevent their proliferation can be determined while factors working on the strategy of the infestation also can be clarified [10]. Parasitic* Lernanthropus* species have a complex life cycle with a number of different larval stages. Between each is a moult when the old cuticle is shed. Pioneer descriptions of life cycle of family Lernanthropidae have been done. In the investigation on* L. kroyeri*, nine larval stages were observed [10]. The stages were named nauplii I, nauplii II, infective copepodid, fixed copepodid I, II, III, and IV, preadult, and adult.

As the species of* Lernanthropus* from cage culture in Setiu Wetland, Terengganu, have been identified as* Lernanthropus latis* [11], obtaining the life cycle details was the main purpose in this research. Within laboratory condition, the salinity and temperature used were maintained the same as at the cage site, where the seabass was taken.

## 2. Materials and Methods

*Lates calcarifer* (seabass), in size ranging from 400 to 600 g, was collected from sea cage culture in Setiu Wetland, Terengganu, Malaysia. The fish were brought back alive to the laboratory of Universiti Malaysia Terengganu for dissection. Adult female* Lernanthropus latis* with eggs were isolated from the seabass gills and placed in a vial containing 20 mL of filtered seawater. The temperature was maintained at 30°C for incubation. Observation was done every hour for detection of nauplii I, nauplii II, and infective copepodid stage. Infection method was applied after all the nauplius moulted to infective copepodid stage. The copepodids in the 20 mL filtered seawater were gently poured into a polytank containing 150 fingerling (2-3 inches in size) in 60 litres of seawater. Dissection was done every five hours for determination of other developmental stages. Drawing was done as soon as the new stage was discovered using camera Lucida which was connected to compound microscope [12].

## 3. Results and Discusson

**Figure 1:**
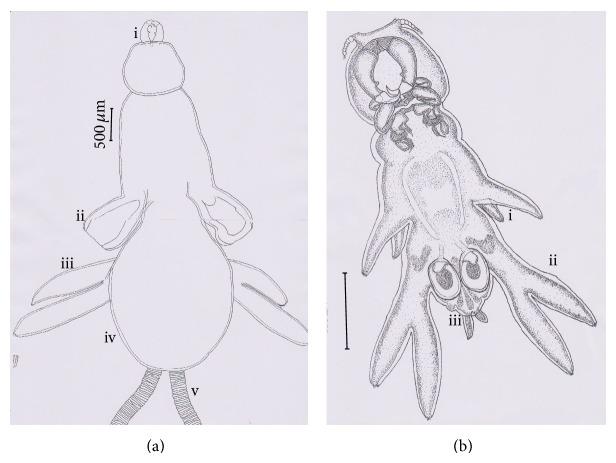
(a) Dorsal view of adult female (scale bar = 500 *μ*m), i: antenna, ii: third leg, iii: fourth leg, iv: dorsal plate, and v: egg string. (b) Ventral view of adult male (scale bar = 500 *μ*m), i: third leg, ii: fourth leg, and iii: genital organ.

Parasite* L. latis* was found attached on the gills of seabass. The adult female clasped their second antennae assisted by third legs to the lamellae-bearing sides of the primary lamellae (gill filaments). The body was positioned between the hemibranchs, with their axis parallel to the lamellae axis [13]. The mature adult female bears two egg strings near the genital segment. The egg string is long and uniseriate; some were coiled with numerous eggs inside the string. Adult female (Figure 1(a)) is bigger than male. Body length is 0.55 to 0.6 cm. The body distinctly consists of two parts: cephalothorax and trunk. Dorsal plate completely widened covering genital organ. Male adult (Figure 1(b)) is smaller than female. Body length is 0.18 to 0.2 cm. Dorsal plate is absent.

**Figure 2:**
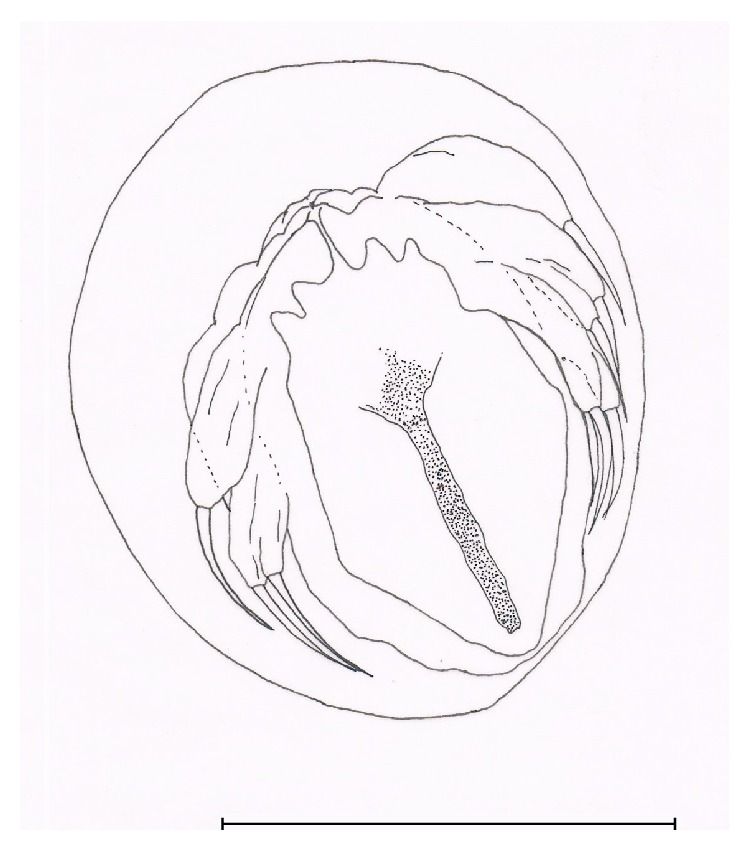
Egg before hatching (scale = 180 *μ*m).

**Figure 3:**
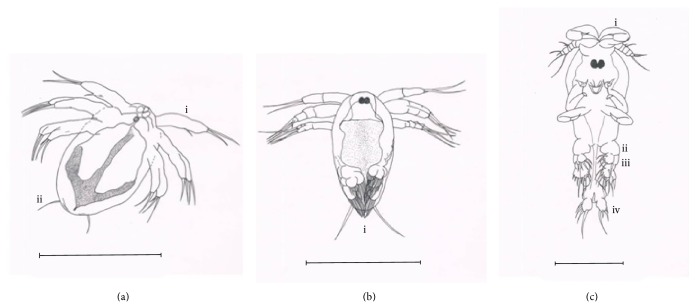
Free living phase of* Lernanthropus latis* life cycle. (a) Nauplii I (scale = 180 *μ*m), i: anterolateral appendages and ii: modified caudal setae. (b) Nauplii II (scale = 180 *μ*m), i: setae of modified legs protruding out of body membrane. (c) Infective copepodid (scale = 110 *μ*m), i: antenna, ii: first leg, iii: second leg, and iv: uropod.

Adult female bears two egg sacs containing 150 to 200 eggs each. The numerous eggs were arranged lengthwise down, where each egg was in its own chamber inside the sac. When the egg is about to hatch, it expands and pushes itself out of the sac membrane (Figure 2). After 15 minutes of observation, the eggs hatch and release nauplii I (Figure 3(a)). The hatching of eggs occured in random and some of them did not hatch at all. The range of body length and width of nauplii I is 1.43 to 1.84 mm and 0.84 to 0.90 mm, respectively, bearing three pairs of anterolateral appendages (Figure 3(a) i) and two modified caudal setae (Figure 3(a) ii) at the posterior of the body. Nauplii II (Figure 3(b)) moulted 15 hours after hatching, also bearing three pairs of anterolateral appendages and two modified caudal setae at the posterior of the body. At this stage, setae of modified legs protrudes out of the body membrane posteriorly (Figure 3(b) i). The range of body length and width is 1.78 to 2.07 mm and 0.80 to 1.00 mm, respectively. Infective copepodid (Figure 3(c)) transformed at 26 to 37 hours after hatching. It swims actively more than nauplii I and nauplii II. Gender is not yet observable at this stage. The body length and width are 2.18 to 2.65 mm and 0.63 to 0.95 mm, respectively. The antenna formation (Figure 3(c) i) is ready for gill attachment. First leg, second leg, and uropod with setae elongated downwards are observable compared to nauplii II stage (Figure 3(c) ii, iii, and iv). All three stages were known as free-swimming stages as they swim randomly without needing a host. The last stage of free swimming, known as infective copepodid, was the stage at the outset of host searching and attachment. Those three stages were named free-living phases.

**Figure 4:**
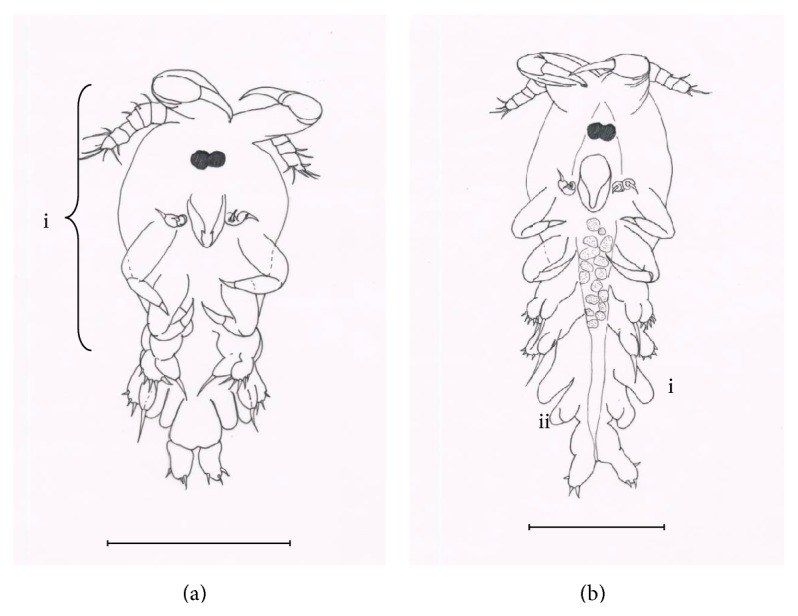
Parasitic phase of* L. latis* life cycle. (a) Fixed copepodid I (scale = 140 *μ*m), i: cephalothorax possesing antennule, antenna, maxillule, mandible, maxilla, maxilliped, and first leg. (b) Fixed copepodid II (scale = 140 *μ*m), i: third leg and ii: fourth leg.

**Figure 5:**
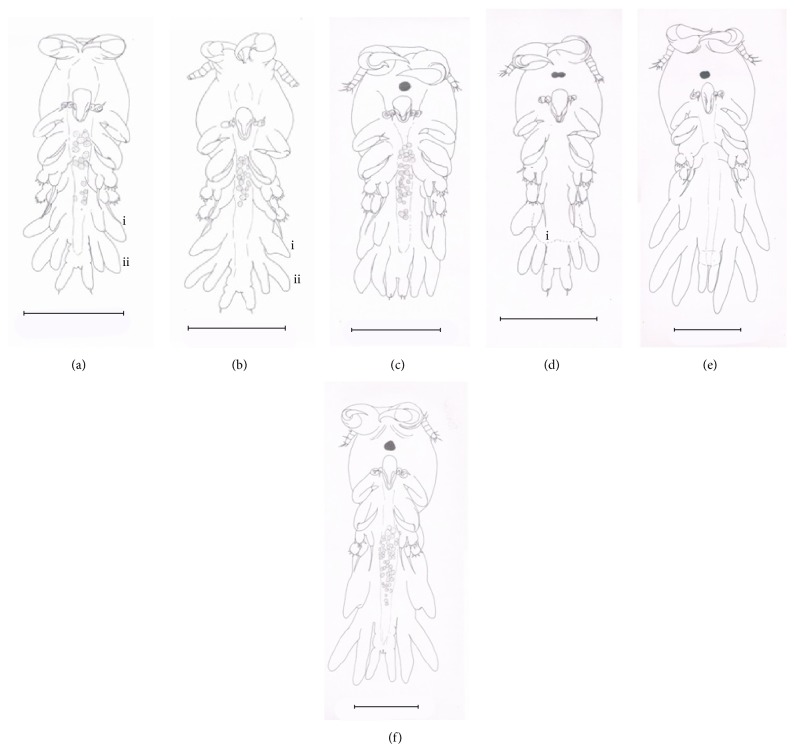
Parasitic phase of* Lernanthropus latis* life cycle. (a) Male fixed copepodid III (scale = 120 *μ*m), i: bifurcated third leg and ii: bifurcated fourth leg. (b) Female fixed copepodid III (scale = 120 *μ*m), i: extended third leg and ii: bifurcated fourth leg. (c) Male fixed copepodid IV (scale = 180 *μ*m). (d) Female fixed copepodid IV (scale = 180 *μ*m), i: formation of dorsal plate. (e) Male preadult (scale = 180 *μ*m). (f) Female preadult (scale = 180 *μ*m).

The remaining stages were collected after infection phase executed. Fixed copepodid I (Figure 4(a)) moults 72 hours after hatching. The gender is not observable. The body length and width are 2.72 to 2.97 mm and 1.06 to 1.07 mm, respectively, cephalothorax being the largest portion of body (Figure 4(a) i). The antennule, antenna, maxillule, mandible, maxilla, maxilliped, and first leg were at the cephalothorax. Fixed copepodid II (Figure 4(b)) moults 84 hours after hatching. The body length and width are 3.2 to 1.05 mm, respectively. The third leg enlarged (Figure 4(b) i). Fourth leg bifurcated forming endopod and exopod (Figure 4(b) ii). The gender is unknown. Fixed copepodid III (Figures 5(a) and 5(b)) moults 95 hours after hatching. The body length and width are 2.97 to 3.74 mm and 1.07 to 1.52 mm, respectively. Gender is distinguishable. Third leg bifurcate for male (Figure 5(a) i) and extend for female (Figure 5(b) i). Bifurcated fourth leg elongated for male and female (Figures 5(a) ii and 5(b) ii). Fixed copepodid IV (Figures 5(c) and 5(d)) moults 109 hours after hatching. The body length and width are 3.9 to 7.44 mm and 1.48 to 3.02 mm, respectively. Third and fourth legs remain in the same shape but are continuously growing for both male and female. Dorsal plate starts to develop for female (Figure 5(d) i). Preadult moult (Figures 5(e) and 5(f)) 207 to 483 hours after hatching. The body length and width are 8.30 to 8.78 mm and 3.12 to 3.36 mm, respectively. Spines present at the tip of third and fourth leg for both male and female. Well formation of gonad is not yet functional for both genders. Dorsal plate keeps on widening for female. The appendages such as antennae, antennule, maxilla, maxillule, maxilliped, first leg, second leg, fourth leg, and genital organ all remain in the same shape but increase in size until reaching adult. As for the third leg, the bifurcation process for male and curve edging for female apparently increase till adult.* Lernanthropus latis* took 482 hours (20 days) to complete the life cycle under laboratory conditions. The salinity and temperature used are 27 ppt and 30°C, respectively. Two-to-four fingerlings were dissected every five hours resulting in the finding of the nine developmental stages as mentioned above. The third leg and dorsal shield are the main parts to differentiate the gender between male and female copepodid. Dorsal shield is absent for male.

The gender was able to be differentiated from the first copepodid (copepodid I) although the larvae were very similar morphologically for both male and female [10]. However, the period of time of larvae moulting from stage to stage and completion the whole life cycle was not stated in the research. In this study, the gender can only be observed at stage of copepodid III. The Families of Caligidae and Lernanthropidae share the same Order, Siphonostomatoida [3, 4]. In the life cycle aspect, both families obtain a similarity in which the first three stages: nauplii I, nauplii II, and copepodid or fixed copepodid (for family Lernanthropidae) are in the phase of free swimming (planktonic). In order to enter the parasitic phase, an infection onto host must be executed. For both* Caligus* and* Lernanthropus*, the stage of copepodid is the main part in which it will start to settle on the host and moult into different stages. Meanwhile, the differentiation can be observed morphologically. The* Caligus* is mostly attached on the skin by use of the frontal filament [14] but the* Lernanthropus* uses the antenna to grip to gill filament and remains at the same location until adult.

Another research on* Lernanthropus latis* life cycle revealed that the time taken by nauplius stage to moult is six to 12 hours, copepodid I is 24 to 36 hours, and the remaining copepodid II, III, IV, V and immature stage was 54 hours, 78 hours, 126 hours, 174 hours, and 222 hours respectively [15]. The nine stages found were similar to the previous study. In the meantime, a significant interaction between water temperature and salinity was detected in determining the success of egg hatching. The best rate of egg hatching was documented within 35‰ salinity and 30°C of temperature. The timing of the different stages of development was directly dependent on water temperature [14]. A research on life cycle of* Caligus rogercresseyi* in rainbow trout was made under natural condition of light and temperature. Five different temperatures were set up at 10.3°C, 12.4°C, 12.8°C, 15.2°C, and 16.7°C. Those temperatures resulted in 45, 31, 32, 26, and 18 days, respectively, for* C. rogercresseyi* to complete a life cycle. From the research, eight developmental stages were observed excluding preadult. The stages were two nauplii, one copepodid, four chalimus, and one adult [14].

Recently, the fish parasites not only play a role in causing fish disease or infecting humans who eat fish but also are used in understanding the fish population. In terms of fish biology, the employment of parasites as biological tags is useful in assessing fish stock and migratory pattern. Knowledge of parasite life cycles may provide information and data for future research.
